# Losing Regulation of the Extracellular Matrix is Strongly Predictive of Unfavorable Prognostic Outcome after Acute Myocardial Infarction

**DOI:** 10.3390/ijms21176219

**Published:** 2020-08-27

**Authors:** Pei-Hsun Sung, Kun-Chen Lin, Han-Tan Chai, John Y. Chiang, Pei-Lin Shao, Chi-Wen Luo, Hon-Kan Yip

**Affiliations:** 1 Division of Cardiology, Department of Internal Medicine, Kaohsiung Chang Gung Memorial Hospital, Chang Gung University College of Medicine, Kaohsiung 83301, Taiwan; e12281@cgmh.org.tw (P.-H.S.); chaiht@mail.cgmh.org.tw (H.-T.C.); 2 Center for Shockwave Medicine and Tissue Engineering, Kaohsiung Chang Gung Memorial Hospital, Kaohsiung 83301, Taiwan; 3 Institute for Translational Research in Biomedicine, Kaohsiung Chang Gung Memorial Hospital, Kaohsiung 83301, Taiwan; 4 Department of Anesthesiology, Kaohsiung Chang Gung Memorial Hospital and Chang Gung University College of Medicine, Kaohsiung 83301, Taiwan; paullin688@gmail.com; 5 Department of Computer Science and Engineering, National Sun Yat-Sen University, Kaohsiung 804201, Taiwan; chiang@cse.nsysu.edu.tw; 6 Department of Healthcare Administration and Medical Informatics, Kaohsiung Medical University, Kaohsiung 80756, Taiwan; 7 Department of Nursing, Asia University, Taichung 413, Taiwan; m8951016@gmail.com; 8 Department of Medical Research, China Medical University Hospital, China Medical University, Taichung 404, Taiwan; 9 Department of Surgery, Kaohsiung Medical University Hospital, Kaohsiung 80756, Taiwan; cwlo0623@gmail.com; 10 Division of Breast Surgery, Department of Surgery, Kaohsiung Medical University Hospital, Kaohsiung 80756, Taiwan

**Keywords:** double knock-out of MMP9 and tPA, acute myocardial infarction, poor prognostic outcome

## Abstract

This study tested the hypothesis that MMP-9^−/−^tPA^−/−^ double knock out (i.e., MT^DKO^) plays a crucial role in the prognostic outcome after acute myocardial infarction (AMI by ligation of left-coronary-artery) in MT^DKO^ mouse. Animals were categorized into sham-operated controls in MT^DKO^ animals (group 1) and in wild type (B6: group 2), AMI-MT^DKO^ (group 3) and AMI-B6 (group 4) animals. They were euthanized, and the ischemic myocardium was harvested, by day 60 post AMI. The mortality rate was significantly higher in group 3 than in other groups and significantly higher in group 4 than in groups 1/2, but it showed no difference in the latter two groups (all *p* < 0.01). By day 28, the left-ventricular (LV) ejection fraction displayed an opposite pattern, whereas by day 60, the gross anatomic infarct size displayed an identical pattern of mortality among the four groups (all *p* < 0.001). The ratio of heart weight to tibial length and the lung injury score exhibited an identical pattern of mortality (*p* < 0.01). The protein expressions of apoptosis (mitochondrial-Bax/cleaved-caspase3/cleaved-PARP), fibrosis (Smad3/T-GF-ß), oxidative stress (NOX-1/NOX-2/oxidized-protein), inflammation (MMPs^2,9^/TNF-α/p-NF-κB), heart failure/pressure overload (BNP/ß-MHC) and mitochondrial/DNA damage (cytosolic-cytochrome-C/γ-H2AX) biomarkers displayed identical patterns, whereas the angiogenesis markers (small vessel number/CD31+cells in LV myocardium) displayed opposite patterns of mortality among the groups (all *p* < 0.0001). The microscopic findings of fibrotic/collagen deposition/infarct areas and inflammatory cell infiltration of LV myocardium were similar to the mortality among the four groups (all *p* < 0.0001). MT^DKO^ strongly predicted unfavorable prognostic outcome after AMI.

## 1. Introduction

Abundant data have revealed that the myocardium of a failing heart (HF), i.e., resulting from myocardial infarction (MI), dilated cardiomyopathy (DCM) or hypertrophic cardiomyopathy (HCM), always undergoes a number of structural alterations, most notably hypertrophy of cardiac myocytes and an increase in extracellular matrix (ECM) proteins [1,2,3,4]. Studies have shown that interstitial fibrosis and excessive accumulation of ECM proteins play crucial roles in regulating LV remodeling [5,6,7,8,9,10,11]. Some studies have further shown that the typical histopathological changes in HCM and MI are characterized by interstitial fibrosis and excessive accumulation of ECM proteins [5,12,13]. Additionally, fibrosis and excessive accumulation of ECM in HCM and MI have been shown to be highly associated with LV dysfunction HF [14,15], i.e., heart failure with reduced ejection fraction (HFrEF), and have been considered as key substrates for ventricular arrhythmias and sudden death [5]. However, the molecular triggers underpinning ECM production are not well established. Intriguingly, a recent study has exhibited that alternation of matrix metalloproteinases (MMPs) was also related to LV remodeling and prognostic outcome in patients with HCM [8]. However, the underlying mechanism of MMPs on LV remodeling and HF events has still not been fully investigated in patients with HCM and MI.

Interestingly, our recent study has demonstrated that the brain infarct volume was remarkably reduced in MMP9 knock out (i.e., MMP9^−/−^) mice as compared with that of wild type (i.e., C57BL/6, simply written as B6) mice in the setting of ischemic stroke (IS) [16]. Additionally, further analysis demonstrated that the underlying mechanism for protecting the brain infarct volume from acute IS is mainly through the accumulated ECM, i.e., tissue remodeling due to deficiency of MMP9, which acts as a proteolytic enzyme, protecting the brain from extensive hemorrhage and transforming the hemorrhagic effect [16]. Furthermore, our previous study [17] showed that MMP9 is the downstream signaling of tissue plasminogen activator (tPA), i.e., tPA is the key mediator for MMP9 activation, and, in turn, acts as the proteolytic enzyme that degrades the ECM so that the endothelial progenitor cell (EPC) can more easily migrate into the distant zone [17,18]. Based on the aforementioned issues, we believe that the accumulated ECM resulting from decreased axis signaling of the tPA and MMP9 pathways might play a critical role in LV remodeling and HFrEF. Accordingly, we created a double knockout (DKO) mouse of MMP9^−/−^tPA^−/−^ to validate our hypothesis.

## 2. Results

### 2.1. Day-3 Mortality Rate and LVEF at Days 0, 14 and 28 after AMI Induction

**Figure 1:**
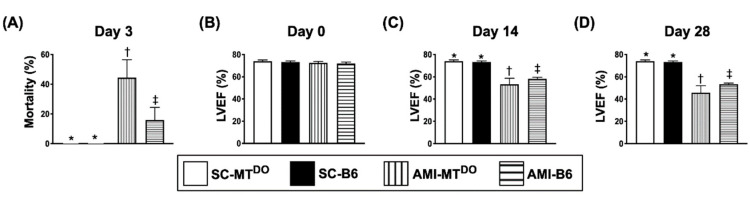
Day-3 mortality rate and left ventricular ejection fraction (LVEF) at days 0, 14 and 28 after acute myocardial infarction (AMI). (**A**) Mortality at day 3 after AMI induction, * vs. other groups with different symbols (†, ‡), *p* < 0.01 (*n* = 20 for each group). (B) LVEF at day 0, *p* > 0.5. (C) LVEF at day 14, * vs. other groups with different symbols (†, ‡), *p* < 0.001. (D) LVEF at day 28, * vs. other groups with different symbols (†, ‡), *p* < 0.001. (*n* = 6 for each group). All statistical analyses were performed by one-way ANOVA, followed by Bonferroni multiple comparison post hoc test. Symbols (*, †, ‡) indicate significance (at the 0.05 level). SC = sham-operated control; MT^DO^ = double knock out of matrix metalloproteinase (MMP)-9 and tissue plasminogen (tPA) in mouse; B6 (i.e., wild type) = C57BL/6 mice.

By day 3 after AMI, the mortality rate was significantly higher in group 3 (AMI-MT^DKO^) than in groups 1 (sham-operated control (SC), i.e., SC-MT^DKO^), 2 (SC-B6) and 4 (AMI-B6) and significantly higher in group 4 than in groups 1 and 2 (40% vs. 15% vs. 0%, *p* < 0.01), but it showed no difference between groups 1 and 2 (Figure 1A).

The LVEF did not differ among the four groups prior to AMI induction (Figure 1B). However, by days 14 and 28 after the AMI procedure (Figure 1C,D), the LVEF was significantly lower in group 3 than in other groups and significantly lower in group 4 than in groups 1 and 2, but it did not differ between groups 1 and 2.

### 2.2. The Ratio of Heart Weight to Tibial Length and the Anatomic Infarct Size by Day 60 after AMI Induction

**Figure 2:**
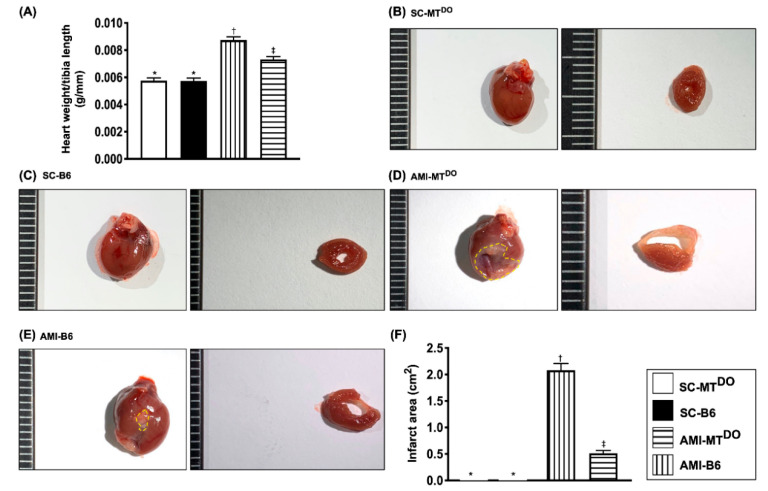
The heart and lung weight and gross anatomical infract area by day 60 after AMI procedure. (**A**) The ratio of heart weight to tibial length, * vs. other groups with different symbols (†, ‡), *p* < 0.01. (**B–E**) Illustrating the morphological features (i.e., the total heart and the cross-section of the heart) of the heart and infarct zone (yellow dotted line) among the four groups. (**F**) Analytical result of gross anatomical infarct area, * vs. other groups with different symbols (†, ‡), *p* < 0.0001. All statistical analyses were performed by one-way ANOVA, followed by Bonferroni multiple comparison post hoc test (*n* = 10 for each group). Symbols (*, †, ‡) indicate significance (at 0.05 level). SC = sham-operated control; MT^DO^ = double knock out of matrix metalloproteinase (MMP)-9 and tissue plasminogen (tPA) in mice; B6 (i.e., wild type) = C57BL/6 mice. AMI = acute myocardial infarction.

The ratio of heart weight to tibial length was significantly increased in group 3 compared to groups 1, 2 and 4 and significantly increased in group 4 compared to groups 1 and 2, but it was similar between groups 1 and 2 (Figure 2A). Additionally, the gross anatomical infarct area displayed a similar pattern to the ratio of heart weight to tibial length among the four groups (Figure 2B–F).

### 2.3. The Protein Expressions of Apoptosis and Fibrosis, and the Lung Injury Score in LV Myocardium by 60 Days after AMI Induction

**Figure 3:**
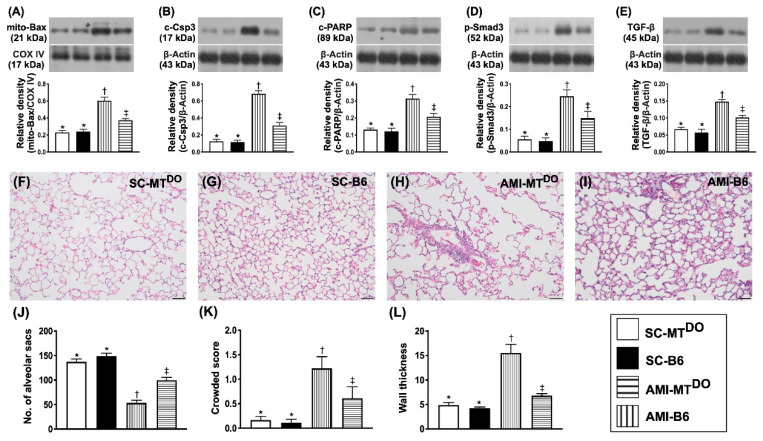
Protein expressions of apoptosis and fibrosis in LV myocardium by 60 days after the AMI procedure. (**A**) Protein expressions of mitochondrial (mito)-Bax, * vs. other groups with different symbols (†, ‡), *p* < 0.0001. (**B**) Protein expression of cleaved caspase 3 (c-Csp3), * vs. other groups with different symbols (†, ‡), *p* < 0.0001. (**C**) Protein expression of cleaved poly (ADP-ribose) polymerase (c-PARP), * vs. other groups with different symbols (†, ‡), *p* < 0.0001. (**D**) Protein expression of phosphorylated (p)-Smad3, * vs. other groups with different symbols (†, ‡), *p* < 0.0001. (**E**) Protein expression of transforming growth factor (TGF)-ß, * vs. other groups with different symbols (†, ‡), *p* < 0.0001. (**F–I**) Illustrating the H&E stain for identification of lung injury score (200×). (**J**) Analytical result of the number of alveolar sacs, * vs. other groups with different symbols (†, ‡), *p* < 0.0001. (**K**) Analytical result of the crowded score, * vs. other groups with different symbols (†, ‡), *p* < 0.0001. (**L**) Analytical result of wall thickness, * vs. other groups with different symbols (†, ‡), *p* < 0.0001. All statistical analyses were performed by one-way ANOVA, followed by Bonferroni multiple comparison post hoc test (*n* = 6 for each group). Symbols (*, †, ‡) indicate significance (at 0.05 level). SC = sham-operated control; MT^DO^ = double knock out of matrix metalloproteinase (MMP)-9 and tissue plasminogen (tPA) in mice; B6 (i.e., wild type) = C57BL/6 mice. LV = left ventricular; AMI = acute myocardial infarction.

The protein expressions of mitochondrial Bax (Figure 3A), cleaved caspase 3 (Figure 3B) and cleaved PARP ((Figure 3C), three indicators of apoptosis, were significantly higher in group 3 than in groups 1, 2 and 4 and significantly higher in group 4 than in groups 1 and 2, but they showed no difference between groups 1 and 2. Additionally, the protein expressions of p-Smad3 (Figure 3D) and TGF-ß (Figure 3E), two indicators of fibrosis, exhibited a similar pattern to apoptosis among the four groups.

It is common sense that advanced left side HF frequently affects the function and integrity of the lung parenchyma. Thus, a haematoxylin and eosin (H&E) stain of the lung specimen was performed. As expected, the number of alveolar sacs was significantly decreased in group 3 compared to other groups and significantly reduced in group 4 compared to groups 1 and 2, but it showed no difference between these latter two groups (Figure 3J). On the other hand, the crowded score (Figure 3K) and wall thickness (Figure 3L) exhibited the opposite pattern to the alveolar sacs.

### 2.4. The Protein Expressions of Oxidative-Stress and Inflammatory Biomarkers in LV Myocardium by 60 Days after AMI Induction

**Figure 4:**
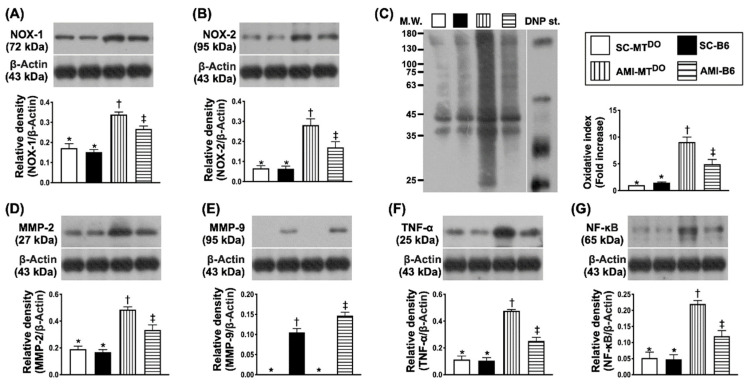
Protein expressions of oxidative-stress and inflammatory biomarkers in LV myocardium by 60 days after AMI procedure. (**A**) Protein expression of NOX-1, * vs. other groups with different symbols (†, ‡), *p* < 0.0001. (**B**) Protein expression of NOX-2, * vs. other groups with different symbols (†, ‡), *p* < 0.0001. (**C**) Oxidized protein expression, * vs. other groups with different symbols (†, ‡), *p* < 0.0001. (Note: left and right lanes shown on the upper panel represent the protein molecular weight marker and control oxidized molecular protein standard, respectively). M.W. = molecular weight; DNP = 1-3 dinitrophenylhydrazone. (**D**) Protein expression of matrix metalloproteinase (MMP)-2, * vs. other groups with different symbols (†, ‡), *p* < 0.0001. (**E**) Protein expression of MMP-9, * vs. other groups with different symbols (†, ‡), *p* < 0.0001. (**F**) Protein expression of tumor necrosis factor (TNF)-α, * vs. other groups with different symbols (†, ‡), *p* < 0.0001. (**G**) Protein expression of phosphorylated nuclear factor (p-NF)-κB, * vs. other groups with different symbols (†, ‡), *p* < 0.0001. All statistical analyses were performed by one-way ANOVA, followed by Bonferroni multiple comparison post hoc test (*n* = 6 for each group). Symbols (*, †, ‡) indicate significance (at 0.05 level). SC = sham-operated control; MT^DO^ = double knock out of matrix metalloproteinase (MMP)-9 and tissue plasminogen (tPA) in mice; B6 (i.e., wild type) = C57BL/6 mice. LV = left ventricular; AMI = acute myocardial infarction.

The protein expressions of NOX-1 (Figure 4A), NOX-2 (Figure 4B) and oxidized protein (Figure 4C), three indices of oxidative stress, were significantly higher in group 3 than in groups 1, 2 and 4, and significantly higher in group 4 than in groups 1 and 2, but they showed no difference between the latter two groups. Additionally, the protein expressions of MMP2 (Figure 4D), MMP9 (Figure 4E), TNF-α (Figure 4F) and p-NF-κB (Figure 4G), four indices of inflammatory biomarkers, revealed an identical pattern of oxidative stress.

### 2.5. The Protein Expressions of Heart Failure/Pressure Overload and Mitochondrial/DNA Damage Biomarkers, and Small Vessel Density in LV Myocardium by 60 Days after AMI Induction

**Figure 5:**
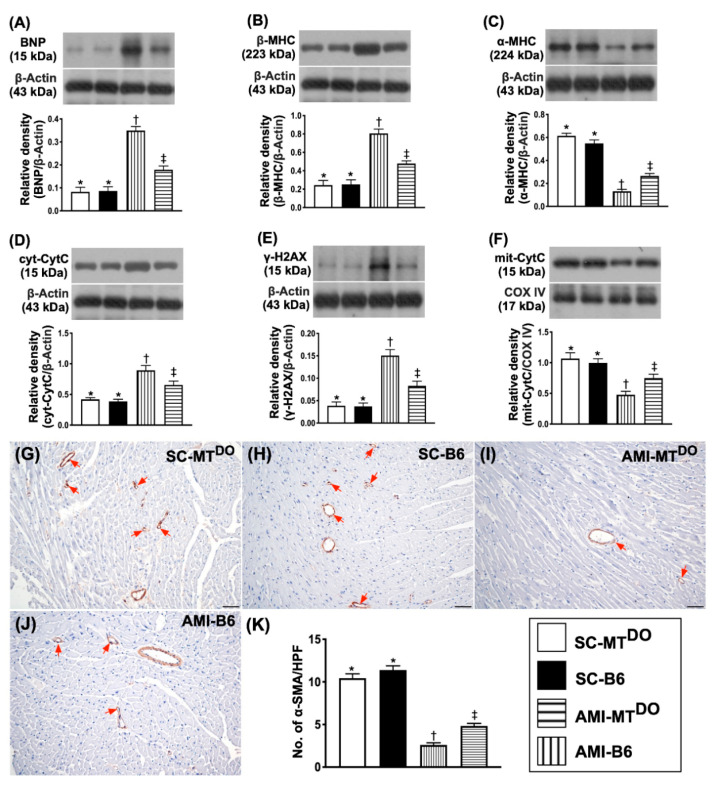
Protein expression of heart failure/pressure overload and mitochondrial/DNA damage biomarkers in LV myocardium by 60 days after the AMI procedure. (**A**) Protein expression of brain natriuretic peptide (BNP), * vs. other groups with different symbols (†, ‡), *p* < 0.0001. (**B**) Protein expression of β-myosin heavy chain (MHC) protein expression, * vs. other groups with different symbols (†, ‡), *p* < 0.0001. (**C**) Protein expression of α-MHC, * vs. other groups with different symbols (†, ‡), *p* < 0.0001. (**D**) Protein expression of cytosolic cytochrome C (cyt-CytoC), * vs. other groups with different symbols (†, ‡), *p* < 0.0001. (**E**) Protein expression of γ-H2AX, * vs. other groups with different symbols (†, ‡), *p* < 0.0001. (**F**) Protein expression of mitochondrial cytochrome C (mit-CytoC), * vs. other groups with different symbols (†, ‡), *p* < 0.0001. (**G–J**) Illustrating the microscopic finding (100−) for identification of small vessel density (red arrows). (**K**) Analytical result of the number of small vessels (i.e., diameter of the vessel ≤ 25 μM), * vs. other groups with different symbols (†, ‡), *p* < 0.0001. The scale bars in the right lower corner represent 100 µm. All statistical analyses were performed by one-way ANOVA, followed by Bonferroni multiple comparison post hoc test (*n* = 6 for each group). Symbols (*, †, ‡) indicate significance (at 0.05 level). SC = sham-operated control; MT^DO^ = double knock out of matrix metalloproteinase (MMP)-9 and tissue plasminogen (tPA) in mouse; B6 (i.e., wild type) = C57BL/6 mice. LV = left ventricular; AMI = acute myocardial infarction.

The protein expression of BNP (Figure 5A), an indicator of heart failure/pressure overload, was significantly higher in group 3 than in groups 1, 2 and 4 and significantly higher in group 4 than in groups 1 and 2, but it did not differ between groups 1 and 2. Additionally, the protein expression of ß-MHC (Figure 5B), an indicator of cardiac hypertrophy, displayed an identical pattern of BNP, whereas the protein expression of α-MHC (Figure 5C), playing an essential role for reversion of cardiac hypertrophy, exhibited the opposite pattern to ß-MHC among the four groups.

The protein expressions of cytosolic cytochrome C (Figure 5D), an index of mitochondrial damage, and γ-H2AX (Figure 5E), an indicator of DNA damage, displayed identical patterns, whereas the protein expression of mitochondrial cytochrome C (Figure 5F), an indicator of mitochondrial integrity, displayed the opposite pattern to BNP among the four groups.

To elucidate the impact of ECM accumulation on the angiogenesis ability, the microscopic finding of α-SMA was performed in the present study. The result showed that the number of small vessels (defined as the diameter of the vessel ≤ 25 μM) was significantly lower in group 3 than in other groups and significantly lower in group 4 than in groups 1 and 2, but this parameter did not differ between groups 1 and 2 (Figure 5G–K), suggesting that deficiency of tPA/MMP9 activity diminished the angiogenesis ability in the ischemic area.

### 2.6. The Fibrosis and Collagen Deposition area, Infarct Area and Cellular Level of DNA Damage Biomarkers in LV Myocardium by 60 Days after AMI Induction

**Figure 6:**
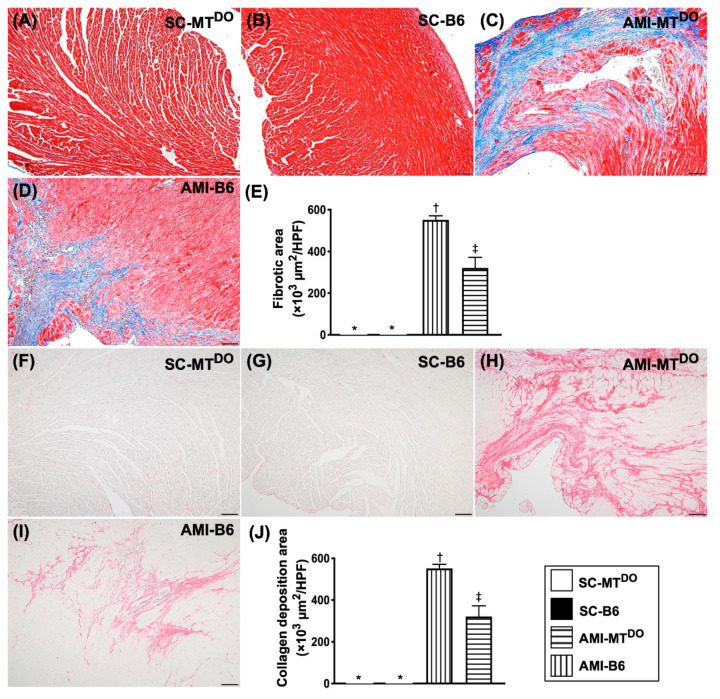
The fibrotic and collagen deposition areas in LV myocardium by 60 days after the AMI procedure. (**A**–**D**) The microscopic findings (100×) of Masson’s trichrome stain for identification of fibrotic areas (blue color). (**E**) Analytic result of the fibrotic areas, * vs. other groups with different symbols (†, ‡), *p* < 0.0001. (**F–I**) The microscopic findings (100×) of Sirius red stain for identification of collagen deposition areas (pink color). (**J**) Analytical result of the collagen deposition areas, * vs. other groups with different symbols (†, ‡), *p* < 0.0001. The scale bars in the right lower corner represent 100 µm. All statistical analyses were performed by one-way ANOVA, followed by Bonferroni multiple comparison post hoc test (*n* = 6 for each group). Symbols (*, †, ‡) indicate significance (at 0.05 level). SC = sham-operated control; MT^DO^ = double knock out of matrix metalloproteinase (MMP)-9 and tissue plasminogen (tPA) in mice; B6 (i.e., wild type) = C57BL/6 mice. LV = left ventricular; AMI = acute myocardial infarction.

The microscopic findings demonstrated that fibrotic (Figure 6A–E) and collagen deposition (Figure 6F–J) areas were significantly higher in group 3 than in groups 1, 2 and 4 and significantly higher in group 3 than in groups 1 and 2, but they did not differ between groups 1 and 2.

**Figure 7:**
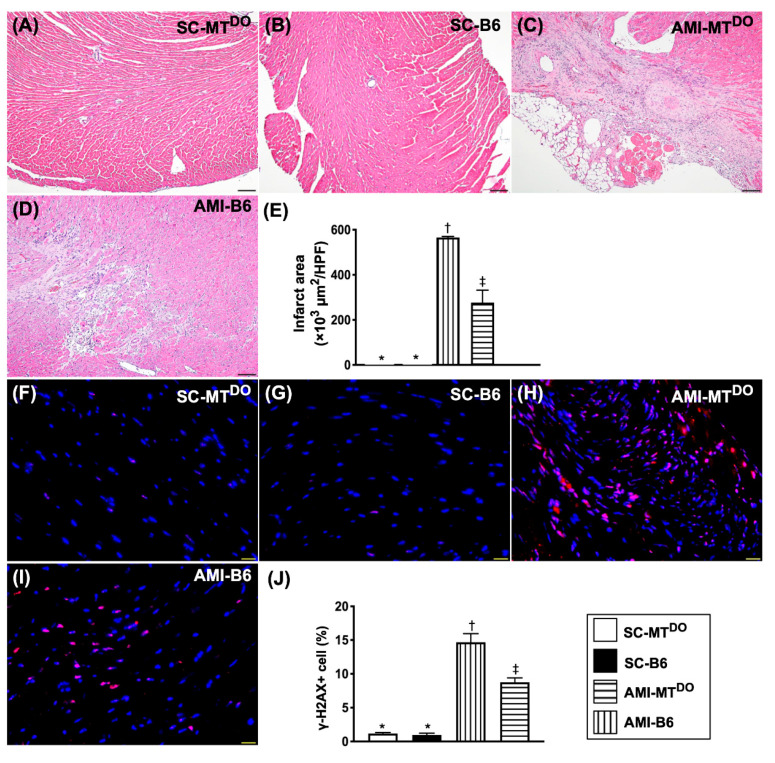
Infarct area and cellular level of DNA damage biomarkers in LV myocardium by 60 days after the AMI procedure. (**A–D**) The microscopic findings (100×) of H.E stain for identification of infarct areas. (**E**) Analytical result of infarct area * vs. other groups with different symbols (†, ‡), *p* < 0.0001. The scale bars in the right lower corner represent 100 µm. (**F–I**) The immunofluorescent (IF) microscopic findings (400×) for identification of positively-stained γ-H2AX+ cells (red color). (**J**) Analytical result of the number of γ-H2AX+ cells. The scale bars in the right lower corner represent 20 µm. All statistical analyses were performed by one-way ANOVA, followed by Bonferroni multiple comparison post hoc test (*n* = 6 for each group). Symbols (*, †, ‡) indicate significance (at 0.05 level). SC = sham-operated control; MT^DO^ = double knock out of matrix metalloproteinase (MMP)-9 and tissue plasminogen (tPA) in mice; B6 (i.e., wild type) = C57BL/6 mice. LV = left ventricular; AMI = acute myocardial infarction.

Consistently, the LV myocardial infarct area (Figure 7A–E) and the γ-H2AX+ cells (Figure 7F–J) exhibited a similar pattern of fibrosis among the four groups.

### 2.7. Inflammatory Cell Infiltration and Endothelial Cell Distribution in LV Myocardium by 60 Days after AMI Induction

**Figure 8:**
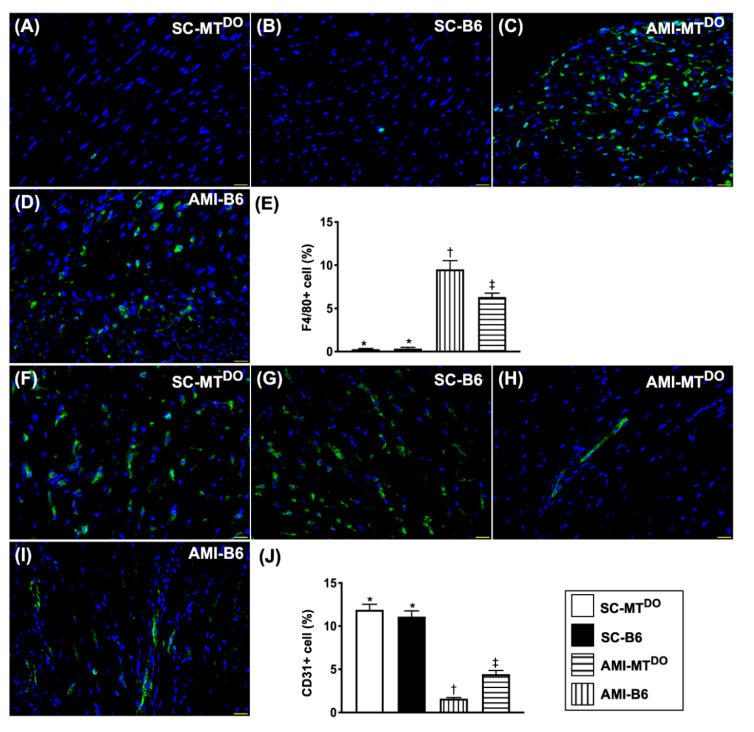
Inflammatory cell infiltration and endothelial cell distribution in the LV myocardium by 60 days after the AMI procedure. (**A–D**) The immunofluorescent (IF) microscopic findings (400×) for identification of F4/80+ cells (green color). (**E**) Analytical result of the number of F4/80+ cells, * vs. other groups with different symbols (†, ‡), *p* < 0.0001. (**F–I)** The IF microscopic findings (400×) for identification of CD31+ cells (green color). (**J**) Analytical result of the number of CD31+ cells, * vs. other groups with different symbols (†, ‡), *p* < 0.0001. The scale bars in the right lower corner represent 20 µm. All statistical analyses were performed by one-way ANOVA, followed by Bonferroni multiple comparison post hoc test (*n* = 6 for each group). Symbols (*, †, ‡) indicate significance (at 0.05 level). SC = sham-operated control; MT^DO^ = double knock out of matrix metalloproteinase (MMP)-9 and tissue plasminogen (tPA) in mouse; B6 (i.e., wild type) = C57BL/6 mice. LV = left ventricular; AMI = acute myocardial infarction.

The cellular expression of F4/80 (Figure 8A–E), an indicator of inflammation, was significantly increased in group 3 compared to groups 1, 2 and 4, and significantly increased in group 4 compared to groups 1 and 2, but it showed similarly between groups 1 and 2. On the other hand, the cellular expression of CD31 (Figure 8F–J), an index of endothelial cell integrity, exhibited the opposite pattern to inflammation among the four groups.

### 2.8. Schematically Proposed Mechanism of tPA/MMP9 Deficiency on the Prognostic Outcome in MT^DKO^ Mice after AMI Induction 

**Figure 9:**
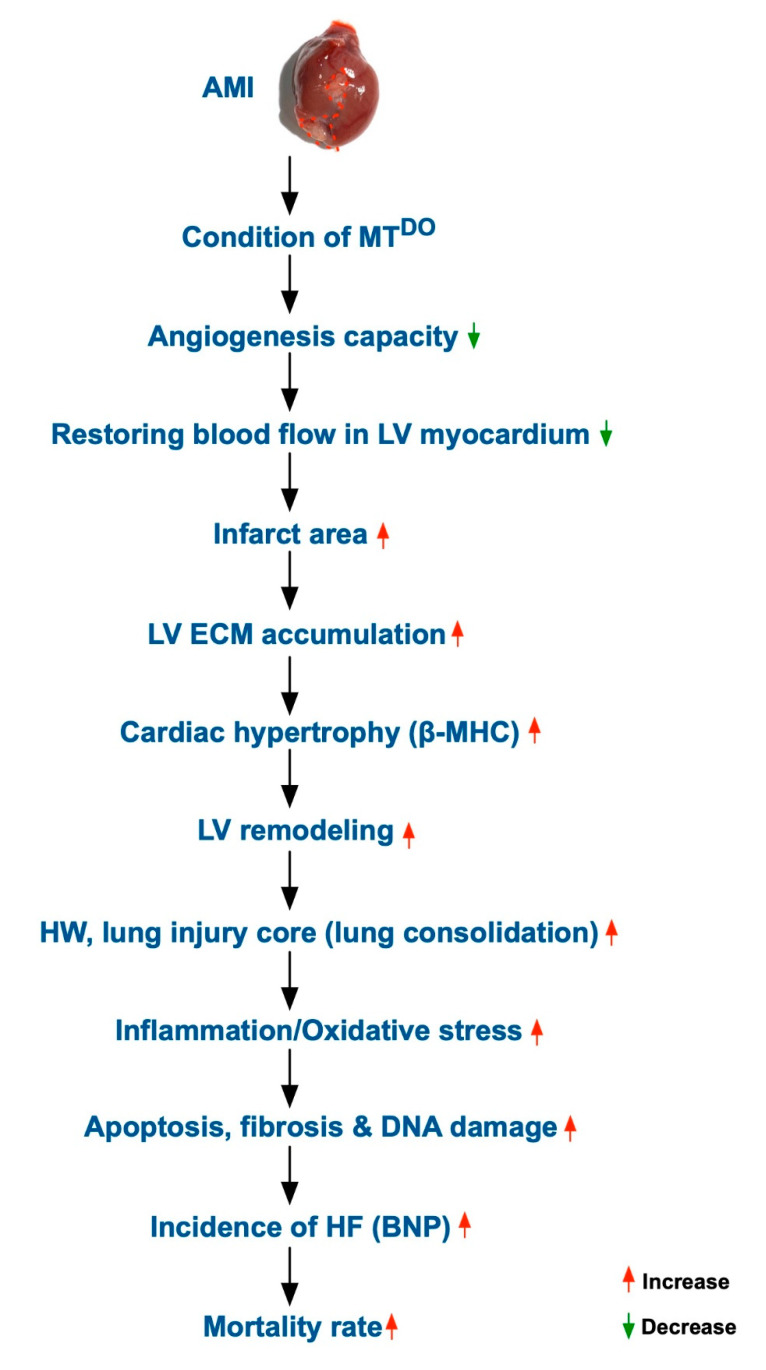
Schematic of the proposed mechanism of the effect of tPA/MMP9 deficiency on the prognostic outcome in MT^DKO^ mice after AMI induction.

Despite the extensive experiments done in the present study, the exact underlying mechanistic basis for how tPA/MMP9 deficiency worsens the prognostic outcome in the MT^DKO^ mice after AMI induction remains uncertain. The proposed mechanism involved in this issue, based on the results of the present study, is illustrated in Figure 9.

## 3. Discussion

This study, which investigated the impact of DKO of MMP9/tPA (i.e., resulted in accumulated ECM) yielded several striking implications. First, the mortality rate after AMI was notably higher in DKO (i.e., MMP9^−/−^tPA^−/−^) mice than in wild type (i.e., B6) mice. Second, the infarct area of LVEF under the identical AMI-induced procedure was significantly larger in DKO mice than in wild type mice. Third, the time points of LEVEF after AMI were remarkably lower in DKO mice than in wild type mice. Finally, the molecular–cellular perturbations were more notably enhanced in DKO mice than in wild type mice.

ECM signaling has been shown to play a critical role in modulating cell fate commitment for cardiovascular tissue engineering [19]. Additionally, changes in the profile and biochemistry of the ECM may be critically involved in the pathogenesis of HFrEF [20]. Furthermore, plentiful clinical data pinpoint a strong association between expansion of the cardiac ECM and adverse outcome in patients with heart failure [20,21,22]. The essential findings in the present study were that as compared with wild type mice, MMP-9^−/−^tPA^−/−^ mice (i.e., a phenomenon of accumulated ECM) had a remarkably larger infarct area (from anatomical and histopathological findings) and lung crowed score, and a markedly reduced LVEF and number of alveolar sacs after AMI induction. These findings could, perhaps, explain why the mortality rate was notably increased in DKO mice than in wild type mice. In this way, our findings acted in concert with the previous studies [19,20,21,22].

An increase in fibrosis and expansion of ECM has also been identified to play a critical role in LV remodeling and HFrEF [19,20,21,23,24]. A principal finding in the present study was that despite the LV infarct area (i.e., indicated myocardial necrosis and lost myocardial mass) being substantially increased in DKO mice than in wild type mice after AMI induction, the heat weight remained significantly higher in DKO mice than in those of wild type mice. Additionally, the IHC stain showed that the collagen deposition area and fibrotic area in the infarct zone were notably larger in DKO mice than in those of the wild type mice. Our findings, in addition to supporting the findings of the previous studies [19,20,21,23,24], further certified that the DKO mice contained many components of ECM proteins as compared with the wild type mice.

Abundant data have revealed that tissue ischemia or necrosis frequently elicited inflammatory reactions and oxidative stress, which, in turn, further damaged the tissues/organs, resulting in ischemia-related organ dysfunction [11,16,17,18,25,26]. Intriguingly, the protein and cellular levels of these aforementioned biomarkers remarkably increased in those of DKO animals compared to those of wild type animals after AMI induction. Additionally, the molecular–cellular perturbations of apoptosis, fibrosis, and mitochondrial and DNA damage markers were also augmented in DKO animals compared to those of wild type animals after AMI induction. Accordingly, our findings, in addition to strengthening the findings of previous studies [11,16,17,18,25,26], could at least in part explain why the LVEF was significantly reduced in DKO animals compared to that of wild type animals after AMI induction.

It is well known that BNP is released in response to volume expansion and increased wall stress of cardiac myocytes. Plentiful evidence has clearly identified that BNP is a sensitive and useful biomarker for detecting HF and prediction of prognostic outcome in HF patients [27,28]. One important finding in the present study was that the protein expression of ß-MHC in the LV myocardium, a biomarker of cardiac hypertrophy, was remarkably increased in DKO mice compared to wild type mice. Additionally, the protein level of BNP in the LV myocardium was significantly upregulated in DKO mice compared to wild type mice. Our findings, in addition to being consistent with the findings of previous studies [27,28], further proved that LV wall stress along with cardiac hypertrophy and pressure overload/HF after AMI induction more easily took place in DKO animals than in wild type animals.

As a matter of fact, the exact reason for why the DKO mice had poorer LVEF and unfavorable outcomes after AMI remained uncertain. A body of evidence has shown that restoration of blood flow in the infarct-related artery is crucial for preserving the LVEF, decreasing the incidence of HF and improving the prognostic outcome in patients after AMI [29,30]. Additionally, our previous studies have identified that tPA was essential for MMP9 activation which, in turn, acts as the proteolytic enzyme for degradation of ECM so that the endothelial progenitor cells (EPCs) can more easily migrate into the distant zone [25,26]. One important finding in the present study was that the number of CD31+ cells (i.e., EPCs/indicator of angiogenesis factor) and the number of small vessels (i.e., an index of angiogenesis/vasculogenesis) in the LV ischemic area were significantly reduced in DKO animals compared to wild type animals. These findings, in addition to being explained by the deficiency of proteolytic enzyme to degrade the ECM [25,26], implied that the blood flow in the infarct area might be more quickly restored in the latter animals than in the former ones. Our findings, in addition to working in concert with the findings of previous studies [29,30], perhaps partially explained the phenomenon observed.

In the present study, we found that tPA deficiency was strongly associated with a decrease in angiogenesis, suggesting that tPA activity plays a key role in angiogenesis/vasculogenesis and restoring the blood flow in ischemic zones [17]. This finding is an extremely relevant piece of clinical data that supports our daily clinical practice to use tPA as a standard method (i.e., thrombolytic therapy) for ST-segment elevation myocardial infarction (STEMI).

## 4. Materials and Methods

### 4.1. Ethics

All animal procedures were approved by the Institute of Animal Care and Use Committee at Kaohsiung Chang Gung Memorial Hospital (No. 2016092701) and performed according to the care and use of laboratory animal guidelines. Animals were kept in an Association for Assessment and Accreditation of Laboratory Animal Care International (AAALAC) approved animal facility in our hospital.

### 4.2. Animal Grouping and Source of Double Knockout Mice (MMP-9^−/−^-tPA^−/−^)

Pathogen-free, adult males of MMP9^−/−^tPA^−/−^ (MT^DKO^) mice, weighing 23–25 g (Charles River Technology, BioLASCO, Taiwan) were categorized into group 1 (sham-operated control (SC) of DKO mice, i.e., SC-MT^DKO^), group 2 ((SC) of wild type (C57BL/6), i.e., SC-B6), group 3 (AMI-MT^DKO^) and group 4 (AMI-B6). 

Mice with gene depletion in both tPA and MMP9 (MMP-9^−/−^-tPA^−/−^, DKO) were generated by crossing tPA knockout (KO) mice with MMP9 KO mice. The genotyping was performed by PCR on tail tip-extracted genomic DNA with primer sets that can specifically detect gene disruptions of tPA and MMP9.

### 4.3. Animal Model of Acute Myocardial Infarction (AMI) Induction 

The procedures of AMI induction from our previous studies were followed [18,26]. In brief, all mice were anesthetized and the heart was exposed via a left thoracotomy under sterile conditions. Sham-operated mice (SC) received the thoracotomy only, while other groups had AMI induced by left coronary artery ligation. Regional myocardial ischemia was evaluated by rapid color change from pinkish to dull reddish over the anterior surface of the LV and rapid development of akinesia and dilatation in the ischemic region. After the procedure, the thoracotomy wound was closed and all mice recovered from anesthesia in a portable animal intensive care unit (ThermoCare^®^) for 24 h.

### 4.4. Functional Assessment by Echocardiography

In each experimental group, transthoracic echocardiography was performed through the Vevo 2100 (Visualsonics, Toronto, ON, Canada) prior to and at days 14 and 28 after different treatments. The M-mode standard two-dimensional (2D) left parasternal long axis echocardiographic examination was conducted. Left ventricular internal dimensions, including left ventricular end-systolic diameter (LVESd) and left ventricular end-diastolic diameter (LVEDd), were measured at the mitral valve and papillary levels of the left ventricle for at least three consecutive cardiac cycles. Left ventricular ejection fraction (LVEF) was evaluated as follows: LVEF (%) = ((LVEDd3-LVESd3)/LVEDd3) × 100%.

### 4.5. Immunohistochemical (IHC) and Immunofluorescent (IF) Staining

The procedure and protocol have been described by previous reports [16,17]. Briefly, sections were incubated with primary antibodies specifically against γ-H2AX (1:500, Abcam, Cambridge, UK), CXCR4 (1:200, Thermo, Waltham, MA, USA), CD31 (1:200, Merck Millipore, Darmstadt, Germany) and F4/80 (1:100, Abcam, Cambridge, UK), while sections incubated with irrelevant antibodies served as controls. Three sections of kidney specimen from each mouse were analyzed. For quantification, three random high-power fields (HPFs) (400× for IHC and IF studies) were analyzed in each section. The mean number of positively-stained cells per HPF for each animal was then determined by summation of all numbers divided by 9.

### 4.6. Western Blot Analysis

Equal amounts (30 µg) of protein extracts were separated by 8–12% SDS-PAGE. After electrophoresis, the separated proteins were transferred onto a polyvinylidene difiuoride (PVDF) membrane (Amersham Biosciences, Amersham, UK). Nonspecific sites were blocked by incubation of the membrane in blocking buffer (5% nonfat dry milk in T-TBS (TBS containing 0.05%Tween 20)) at room temperature for one hour. Then, the membranes were incubated with the indicated primary antibodies (matrix metalloproteinase (MMP)-2 (1:1000, Cell Signaling, Beverly, MA, USA), MMP-9 (1:1000, Abcam), phosphorylated (p)-nuclear factor (NF)-κB (1:1000, Cell Signaling), tumor necrosis factor (TNF)-α (1:1000, Cell Signaling), cleaved caspase 3 (1:1000, Cell Signaling), mitochondrial Bax (1:1000, Abcam), mitochondrial Bax (1:1000, Abcam), cleaved caspase 3 (1:1000, Cell Signaling), cleaved poly (ADP-ribose) polymerase (C-PARP) (1:1000, Cell Signaling), phosphorylated (p)-Smad3 (1:1000, Cell Signaling), transforming growth factor (TGF)-ß (1:1000, Abcam), NOX-1 (1:1500, Sigma, Darmstadt, Germany), NOX-2 (1:1000, Sigma), brain natriuretic peptide (BNP) (1:1500, Abcam), ß-myosin heavy chain (ß-MHC) (1:1000, Santa Cruz, Dallas, TX, USA), α-MHC (1:1000, Santa Cruz), cytosolic cytochrome C (1:2000, BD, Franklin Lakes, NJ, USA), mitochondrial cytochrome C (1:2000, BD), phosphorylated (p)-γ-H2AX (1:1000, Cell Signaling) and GAPDH (I: 10000, Cell Signaling)) for 1 h at room temperature. Horseradish peroxidase-conjugated anti-rabbit or anti-mouse immunoglobulin IgG (I:2000, Cell Signaling) was used as a secondary antibody for one-hour incubation at room temperature. After being washed, the immunoreactive membranes were visualized by enhanced chemiluminescence (ECL; Amersham Biosciences, Amersham, UK) and were exposed to medical X-ray film (FUAJI).

### 4.7. Histological Study of Fibrosis and Collagen-Deposition Area 

The procedure and protocol have been described in our previous report [25]. In detail, Masson’s trichrome and Sirius red staining were used for studying fibrosis and collagen deposition in LV myocardium and quadriceps muscle. Three 4 µm thick serial sections of LV myocardium were prepared by Cryostat (Leica CM3050S). The integrated area (µm^2^) of fibrosis in each section was calculated using Image Tool 3 (IT3) image analysis software (University of Texas, Health Science Center, San Antonio, UTHSCSA; Image Tool for Windows, Version 3.0, USA). Three selected sections were quantified for each animal. Three randomly selected HPFs (100×) were analyzed in each section. After determining the number of pixels in each fibrotic area per HPF, the numbers of pixels obtained from the three HPFs were summated. The procedure was repeated in two other sections for each animal. The mean pixel number per HPF for each animal was then determined by summating all pixel numbers and dividing by 9. The mean integrated area (µm^2^) of fibrosis in LV myocardium per HPF was obtained using a conversion factor of 19.24 (1 µm^2^ represented 19.24 pixels).

### 4.8. Vessel Density in Myocardial and Limb Ischemic Areas

The procedure and protocol have been described in our previous report [25]. In detail, immunohistochemical (IHC) staining of blood vessels was performed (*n* = 6 for each group) with α-smooth muscle actin (SMA) (1:400) as primary antibody at room temperature for 1 h, followed by washing with PBS three times. Ten minutes after the addition of the anti-mouse-HRP conjugated secondary antibody, the tissue sections were washed with PBS three times. Then 3,3′ diaminobenzidine (DAB) (0.7 gm/tablet) (Sigma) was added, followed by washing with PBS three times after one minute. Finally, hematoxylin was added as a counter-stain for nuclei, followed by washing twice with PBS after one minute. Three sections of quadriceps were analyzed in each mouse. For quantification, three randomly selected HPFs (100×) were analyzed in each section. The mean number per HPF for each animal was then determined by summation of all numbers divided by 9.

### 4.9. Assessment of Oxidative Stress

The procedure and protocol for assessing the protein expression of oxidative stress have been detailed in our previous reports [31,32]. The Oxyblot Oxidized Protein Detection Kit was purchased from Chemicon, Billerica, MA, USA (S7150). DNPH derivatization was carried out on 6 μg of protein for 15 min according to the manufacturer’s instructions. One-dimensional electrophoresis was carried out on 12% SDS/polyacrylamide gel after DNPH derivatization. Proteins were transferred to nitrocellulose membranes, which were then incubated in the primary antibody solution (anti-DNP 1: 150) for 2 h, followed by incubation in secondary antibody solution (1:300) for 1 h at room temperature. The washing procedure was repeated eight times within 40 min. Immunoreactive bands were visualized by enhanced chemiluminescence (ECL; Amersham Biosciences, Amersham, UK), which was then exposed to Biomax L film (Kodak, Rochester, NY, USA). For quantification, ECL signals were digitized using Labwork software (UVP, Waltham, MA, USA). For Oxyblot protein analysis, a standard control was loaded on each gel.

### 4.10. Pathological Assessment of Lung Injury 

The procedure and protocol have been described in our previous reports [19,20]. In detail, lung specimens were sectioned at 5 µm for light microscopy and H&E staining was performed to investigate the number of alveolar sacs in a blinded fashion. Three lung sections from each mouse were analyzed and three randomly selected HPFs (×200) were examined in each section. The mean number per HPF for each animal was then determined by summation of all numbers divided by 9. The extent of crowded area, which was defined as the region of thickened septa in lung parenchyma associated with partial or complete collapse of alveoli on H & E-stained sections, was also determined in a blinded fashion. The following scoring system [31,32] was adopted: 0 = no detectable crowded area; 1 = <15% of crowded area; 2 = 15–25% of crowded area; 3 = 25–50% of crowded area; 4 = 50–75% of crowded area; 5 = >75–100% of crowded area/HPF.

### 4.11. Statistical Analysis

Quantitative data were expressed as mean ± standard deviation (SD). Statistical analysis was performed by ANOVA followed by Bonferroni multiple comparison post hoc test. Statistical analysis was performed using SAS statistical software for Windows version 8.2 (SAS institute, Cary, NC, USA). A probability value of less than 0.05 was considered as statistically significant.

## 5. Conclusions

In conclusion, the results of the present study demonstrated that after AMI induction, MMP9^−/−^-tPA^−/−^ mice had larger infarction size, lower LVEF and higher mortality than wild type mice. 

Study limitation: This study has limitations. First, although the results were attractive and promising, the underlying mechanism for why the DKO mice had poorer prognostic outcomes after AMI than in those wild type mice remains uncertain. Accordingly, Figure 9 was plotted based on our findings to schematically illustrate the proposed mechanism underlying the causes that DKO mice had unfavorable prognostic outcomes after AMI. Second, the study period was only two months; thus, whether the long-term outcome is still unfavorable in DKO animals compared to wild type animals or vice versa is currently unclear. Third, without an animal model of single gene KO of AMI (i.e., either tPA or MMP9), we did not know whether a single gene KO on its own has an effect. This raises the hypothesis that there are other target pathways that these genes are involved in.
